# Tolerogenic Dendritic Cells: The Pearl of Immunotherapy in Organ Transplantation

**DOI:** 10.3389/fimmu.2020.552988

**Published:** 2020-10-06

**Authors:** Quan Zhuang, Haozheng Cai, Qingtai Cao, Zixin Li, Shu Liu, Yingzi Ming

**Affiliations:** ^1^Transplantation Center of the 3rd Xiangya Hospital, Central South University, Changsha, China; ^2^Research Center of National Health Ministry on Transplantation Medicine, Changsha, China; ^3^Hunan Normal University School of Medicine, Changsha, China

**Keywords:** organ transplantation, dendritic cell, tolerogenic dendritic cell, immune tolerance, metabolism

## Abstract

Over a half century, organ transplantation has become an effective method for the treatment of end-stage visceral diseases. Although the application of immunosuppressants (IS) minimizes the rate of allograft rejection, the common use of IS bring many adverse effects to transplant patients. Moreover, true transplant tolerance is very rare in clinical practice. Dendritic cells (DCs) are thought to be the most potent antigen-presenting cells, which makes a bridge between innate and adaptive immunity. Among their subsets, a small portion of DCs with immunoregulatory function was known as tolerogenic DC (Tol-DC). Previous reports demonstrated the ability of adoptively transferred Tol-DC to approach transplant tolerance in animal models. In this study, we summarized the properties, ex vivo generation, metabolism, and clinical attempts of Tol-DC. Tol-DC is expected to become a substitute for IS to enable patients to achieve immune tolerance in the future.

## Introduction

Since Dr. Joseph Murray performed the first successful renal operation between identical twins in 1954, organ transplantation has developed extensively (1). However, transplant surgeons and immunologists around the world are always looking for better and safer treatment for severe intra- or post-transplant complications, including rejection, tumor, and infection, which directly or indirectly result from the allograft itself or application of immunosuppressive agents (2). Moreover, traditional immunosuppressants (IS) commonly focus on adaptive immunity (T and B cells); however, once they are activated, stalling the rejection process becomes considerably difficult (3). Therefore, understanding the various factors that activate T and B cells is significant to the therapies for anti-rejection. Dr. Ralph Steinman in 1973 first described dendritic cells (DC) (4). DC are considered to be the most potential antigen-presenting cells (APC), which recognize non-self or even self-antigen and stimulate powerful adaptive-immune cells, such as effector or memory T cells (Teff or Tmem) and indirectly induce plasma cells for antibody production (5). Depleting DC seems to be very effective and technologically advanced for the prevention of organ transplant rejection, which can result in surprising immunodeficiency and lead to some unexpected issues in the body (6). Hence, modifying the DC phenotype and function for inducing transplant tolerance is necessary. A recent study shows prospective strategies to minimize drug treatment, and a reduction in rejection was achieved by combining reduced amounts of IS with immunoregulatory cell therapy in solid organ transplantation (7). Additionally, many reports focus on cell therapy in organ transplantation, including mesenchymal stem cells (MSC), regulatory macrophages (Mreg), tolerogenic dendritic cells (Tol-DC), and regulatory T (Treg) and B (Breg) cells (8–11). Herein, our attention is focused on Tol-DC, which show immunoregulatory functions in autoimmune diseases (12), infections (13), and cancers (14) as well as organ transplant issues (8). We review their features, ex vivo generation, and clinical applications and discuss their diverse effects on organ transplantation.

## The Characteristics and Biomarkers of Tol-DC

DC, which are the so-called professional APC, characterize the bridge to development of an adaptive immune response (specific cell- and antibody-mediated clearance) from the innate immune response (15). DC were first distinguished in lymphoid tissues from other leukocytes on the basis of this idiosyncratic cell shape and an absence of critical lymphocyte and phagocyte properties (16) and subsequently identified in essentially all other tissues of the body. Immediately after transplantation, pattern recognition receptor (PRR)-mediated danger signals activate DC, leading to APC maturation, upregulation of costimulatory molecules, and secretion of proinflammatory cytokines and cytotoxicity (17). At the present time, four main cell types are generally classified as DC: conventional DC (cDC), plasmacytoid DC (pDC), Langerhans cells, and monocyte-derived DC (mono-DC). Among solid organ transplant models, according to the three allorecognition pathways (direct, indirect, and semidirect pathways), DC either derived from donor or recipient tissues and carrying donor major histocompatibility complex (MHC)-specific antigens could be recognized in the secondary lymphatic tissues of recipients to activate a T cell alloimmune response (18). Nonetheless, in addition to the rejection contribution, DC also play an essential role in allograft tolerance, which shows DC in transplanted models have two sides (19–21). Some DC that are able to suppress immune responses are initially termed as Tol-DC.

Mature DC exhibit the characteristics of high expression of the surface MHC-II and costimulatory molecules (CD80/CD86 and CD40). On the contrary, Tol-DC are often characterized by low expression of MHC-II and CD80/CD86 and CD40, termed as a state of “semi-maturity” (8). Additionally, Tol-DC are also featured with increased expression of anti-inflammatory molecules, such as interleukin-10 (IL-10) and transforming growth factor-beta (TGF-β), and decreased levels of IL-12p70 and other proinflammatory cytokines (22).

Transcriptome and proteome studies illustrate distinctive molecular signatures of Tol-DC. Though it is still difficult to find uniform surface markers to define Tol-DC, it is reported that some genes, such as CYP24A1, MUCL1, MAP7, CCL18, C1QB, C1QC, CYP7B1, and CNGA1, could be considered as possible biomarkers for Tol-DC (23).

There are also other molecules that can be regarded as the biomarkers of Tol-DC. The complement subunit C1q was recently identified as a biomarker for monocyte-derived Tol-DC, which could suppress CD4^+^ T-cell activation *via* increasing IL-10 secretion (24). Immature DC are a rich source of active C1q, and the expression of C1q is downregulated when DC are approaching the mature state (25). Globular C1q receptors (gC1qR) are one of the receptors expressed in the surface of mono-DC, and C1q could inhibit the differentiation of DC from its precursor *via* combination with gC1qR and DC-specific intercellular-adhesion-molecule-3 grabbing non-integrin (DC-SIGN) (26). In addition, C1q is a functional ligand for leukocyte-associated Ig-like receptor 1 (CD305), which is a transmembrane protein expressed on both myeloid and lymphoid cells, restricting DC differentiation and activation (27). In the immunotherapy of pollen allergic patients, the increased levels of C1q expressed by Tol-DC in peripheral blood mononuclear cells (PBMC) represent a candidate biomarker of early efficacy of allergen immunotherapy (28, 29). Macrophage inhibitor cytokine (MIC-1) is a divergent member of the TGF-β superfamily, and the high expression of MIC-1 has been observed in Tol-DC (30).

Traditionally, the everlasting immaturity of DC is conducive to the tolerant consequence (31). Recent studies, nonetheless, show that, in some cases, mature DC could also display the characteristic of tolerance. For instance, stimulation by recombinant soluble *Schistosoma mansoni* egg antigen (rSm29) could induce mono-DC with high expression of MHC-II and costimulatory molecules while rSm29 could increase IL-10 level and decrease levels of IL-12p40 and interferon-gamma (IFN-γ) in cultured mono-DC, which results in a great therapeutic efficacy on cutaneous leishmaniasis (32).

## The Ex Vivo Induction of Tol-DC

**Figure 1:**
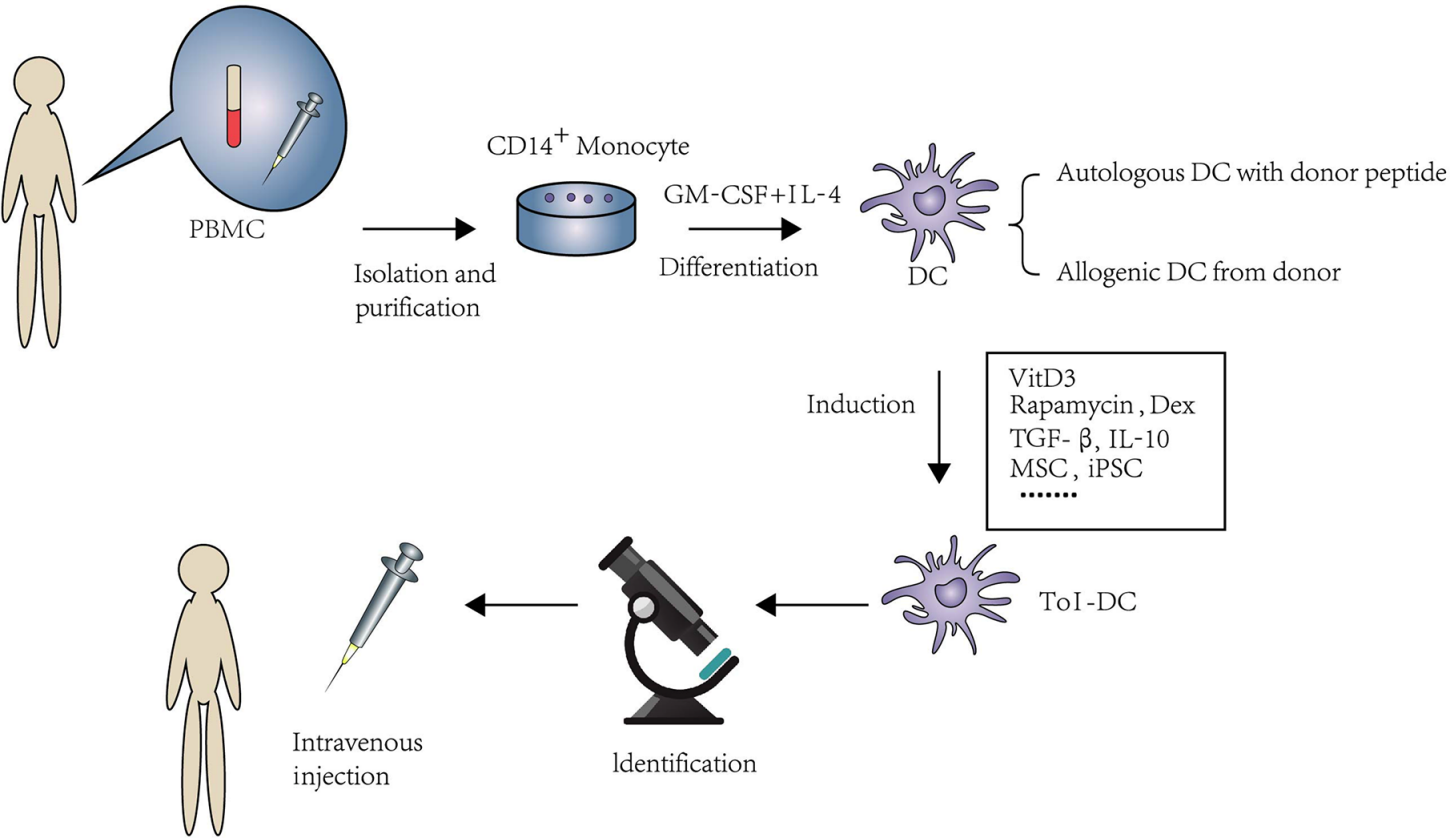
An overview of isolation and expansion procedures of Tol-DC from PBMC and their administration in clinical approaches. PBMC are the source of DC in human. CD14+monocytes were extracted by immunomagnetic separation. Under the stimulation of GM-CSF and IL-4, monocytes were differentiated to DC. The source of DC can be from either donor-derived or autologous DC loaded with donor peptide. Tol-DC can be induced by clinical approved drugs, cytokines, experimental inhibitors, and genetic engineering or biological intervention. After purification and identification. Tol-DC can be transferred to the potential patients through intravenous injection.

Large amounts of DC can be obtained from monocytes pulsed by granulocyte/macrophage colony-stimulating factor (GM-CSF) and IL-4 (33, 34). In rodents, DC are derived from bone marrow cells; nonetheless, DC are usually derived from peripheral blood mononuclear cell (PBMC) in human. The reason why monocytes are considered as the source of DC is that they are easily obtained and more abundant than other DC precursors. Generally, DC can be induced to immunologic DC and Tol-DC *via* different stimulation *in vitro*. There is currently many a protocol to induce Tol-DC ex vivo. Usually, protocols of Tol-DC induction need diverse stimulators and technology, such as clinically approved drugs, cytokines, experimental inhibitors, and genetic engineering or biological intervention. The process of the generation of Tol-DC is summarized in **Figure 1**.

### Clinical Drugs

Vitamin D3 (VitD3) is a fat-soluble hormone that can be acquired from food or be biosynthesized in the skin upon ultraviolet-B radiation and is commonly applied as a drug for rickets, which is considered to be one of the most commonly used strategies for inducing Tol-DC *in vitro*. VitD3-Tol-DC show low expression of MHC-II, CD80, CD86, and CD40 and high secretion or expression of IL-10, indoleamine-2,3-dioxygenase (IDO), and even immunoglobulin-like transcript 3 (ILT3) (35–38). Several inflammatory pathways are involved in this process, such as extracellular signal-regulated kinase (ERK) 1/2 signaling cascade and specificity protein 1 (SP1) signaling factor and nuclear factor-kappa B (NF-κB) (23). A recent study indicates that high expressions of both MAP7 and MUCL1 genes are observed in VitD3-Tol-DC (39).

Immunosuppressants (IS) are also commonly used to induce Tol-DC *in vitro*. IS, such as rapamycin and dexamethasone (Dex), are proven to be effective for Tol-DC induction *in vitro*. Rapamycin (mTOR inhibitor) could suppress DC maturity with intermediate levels of MHC-II and costimulatory molecules (40). Campos-Acuña et al. transferred Tol-DC conditioned by rapamycin and activated by mono-phosphoryl lipid A to a murine skin graft model, resulting in a longer allograft survival period, more Treg proliferation, and cytokine pattern modification (41). Dex is a steroid widely used for the prevention and treatment of organ rejection. Polymeric nanoparticles containing ovalbumin (OVA) and Dex could change DC to Tol-DC phenotype, which could profoundly suppress OVA-specific immune responses *in vivo* (42). Tol-DC conditioned by Dex with a cocktail of cytokines (IL-1β, IL-6, TNF-α, and prostaglandin E2 (PGE2)) was tested in a clinical trial to evaluate the safety of Tol-DC in the treatment of refractory Crohn’s disease (CrD) (43). Human monocyte-derived Tol-DC generated from Dex and VitD3 exhibit a typical tolerogenic phenotype of reduced costimulatory molecules and low production of proinflammatory cytokines (44). This protocol was also used to treat rheumatoid arthritis patients (45).

### Cytokines

There are several cytokines used to induce Tol-DC *in vitro*, and most use IL-10 and TGF-β. Under the stimulation of IL-10, the expression of MHC-II and costimulatory molecules in DC could be reduced (46). There are two subpopulations of IL-10-pulsed DC: CD83^high^CCR7^+^HLA-DR^high^IL-10^+^ DC and CD83^low^CCR7^+^HLA-DR^low^IL-10^+^ DC. The former may become a promising choice for induction or restoration of tolerance *in vivo* because of their stable tolerogenic phenotype, even stimulated by inflammatory molecules, and they could induce highly potent Treg (47). TGF-β increases the expression of programmed death-ligand 1 (PD-L1) on DC, induced T cell apoptosis, and enhanced Treg differentiation (48). Moreover, TGF-β secreted by endothelial stromal cells could induce high expression of Fas-ligand (FasL) in Tol-DC through the ERK pathway (49). Compared to Dex, rapamycin, and TGF-β, IL-10 could induce stronger Tol-DC. Therefore, IL-10 seems to be the optimal inducible therapy for some immune diseases (50). In addition to IL-10 and TGF-β, there are also other cytokines that could induce Tol-DC *in vitro*, such as MIC-1, tumor necrosis factor α (TNF-α)-induced protein 8 like-1 (TIPE1) and PGE2. The expression of malat-1 circular RNA (circ_Malat 1) is the mature signal of DC. When treated with recombinant MIC-1 *in vitro*, the expression of surface molecules CD83, CD86, and HLA-DR is suppressed in DC as a result of the inhibition of circ_Malat 1 and NF-κB pathways. TIPE1, a new member of the TNF-α-induced protein 8 family, could boost PD-L1 expression on DC and restrain the signal transduction to T cell activation (51). Mature DC induced by PGE2 could produce IDO and promote immunoregulatory capacity (52). Moreover, Tol-DC generated by Dex and a maturation cocktail composed of IL-1β, IL-6, TNF-α, and PGE2 could express more E-type prostanoid (EP) receptors 2 and 3, which, activated by PGE2, can induce IL-10 secretion, exhibiting their tolerant function (53).

### Inhibitors/Activators of NF-κB and STAT

NF-κB is a family of dimeric transcription factors (54), and the maturity of DC is related to the activation of NF-κB (55). LF 15-0195 (LF) is a chemically synthesized analog of the immunosuppressant 15 deoxyspergualin, which possesses higher immunosuppressive activity. It is also a blocker of NF-κB. LF-treated DC are characterized by low expression of MHC-II, CD80, CD86, and high expression of anti-inflammatory molecules. These Tol-DC increase CD4^+^CD25^+^CTLA4^+^ and FOXP3^+^Treg levels and improve cardiac graft survival (56). RelB is one of the NF-κB subunits. Tol-DC could be acquired *via* silencing RelB using small interfering RNA, and this kind of Tol-DC also prolongs the survival of the cardiac graft through promoting the induction of Treg (57). NF-κB inhibitors in the induction of Tol-DC has already been applied in clinical trials. In a clinical trial on rheumatoid arthritis, Tol-DC were induced by Bay11-7082, the inhibitor of NF-κB, which irreversibly inhibited NF-κB by preventing phosphorylation of IκBa (58).

Signal transducer and activator of transcription (STAT) is essential in the development and maturation of DC. A total of seven STAT proteins have been identified (STAT1, STAT2, STAT3, STAT4, STAT5a, STAT5b, STAT6) (59). The inhibition or activation of different STAT signals may regulate the phenotype of DC. STAT1 and STAT2 are important in the activation of DC. STAT1 is required for the increased expression of costimulatory molecules in DC (60). Following the stimulation by IFN-γ, the activation of STAT1 could promote the maturation of DC. However, to inhibit the activation of STAT1 *in vitro* by flavonoids, the expression of PD-L1 is decreased in DC, and DC are tend more to an immature phenotype (61). When STAT1 is silenced in inflammation-stimulated DC by siRNA, the expression of CD83 and CD86 are also decreased, and the expression of anti-inflammatory molecules are increased (62). Similarly to STAT1, STAT2 is required for the activation and cross-presentation of DC under the stimulation of toll-like receptor (TLR) ligands (63).

Compared to STAT1 and STAT2, STAT3 is considered to be the negative inhibitor of DC. The activation of STAT3 induces the tolerogenicity in DC, whereas the inhibition of STAT3 induces matures DC. Human DC treated with IFN-α are characterized by high expression of PD-L1 and decreased production of IL-12. However, IFN-α-induced PD-L1 expression is downregulated by inhibitors of p38, Jak, and STAT3 (64). STAT3-deficient DC could enhance immune activity, including increased proinflammatory cytokine production, antigen (Ag)-dependent T cell activation, and resistance to IL-10–mediated suppression (65). The tolerogenicity of DC is correlated to the activation of STAT3. Thymic stromal lymphopoietin (TSLP) can induce the activation of DC with high expression of costimulatory and proinflammatory molecules. STAT5 is required for TSLP-dependent activation, which is a critical component for the promotion of Th2 response immunity during airway inflammation (66). JQ1 is an inhibitor of STAT5. When LPS-activated DC are treated with JQ1, STAT5 phosphorylation and nuclear accumulation is inhibited. As a result of the prevention of STAT5, the expression of CD83 in LPS-DC and the level of IL-12p70 released by DC are decreased (67). The activation of STAT5 may have connection with the maturation of DC followed by external stimulus.

In conclusion, the expression of NF-κB And STAT is critical in the induction of Tol-DC. The inhibition of STAT1, STAT2, and STAT5, but not the activation of STAT3, contribute to the induction of Tol-DC.

### Genetic Engineering and Biological Intervention

There are also other protocols that can induce Tol-DC *in vitro*. Strategies of genetic engineering have also been used to induce Tol-DC, including gene knockout, knockdown, and transgenic over-expression of dominant active or negative mutants of molecules (68). For example, promoting the expression of IL-10-related genes in DC could attenuate liver fibrosis in mice *via* increasing Treg induction. This kind of IL-10^+^DC is characterized by low expression of costimulatory molecules (69). Nuclear paraspeckle assembly transcript 1 (NEAT1) is proven to use NACHT, LRR, and PYD domain-containing protein 3 (NLRP3) inflammasomes as molecular decoys for miR-3076-3p, so knockdown NEAT1 could facilitate the tolerogenic phenotype in DC, which prevents progression of experimental autoimmune myocarditis and induces immune tolerance in a heart transplantation model (70). The metastasis associated in lung adenocarcinoma transcript 1 (MALAT1) overexpression promotes DC-SIGN expression by functioning as an miR155-5p sponge in the DC cytoplasm, which derives DC to Tol-DC with low expression of costimulatory molecules and high IL-10 secretion, protecting mice from acute rejection after cardiac transplantation (71). Apart from these, some biological interventions have also been used to induce Tol-DC, such as mesenchymal stem cells (MSCs) (72), induced pluripotent stem cells (iPSCs) (73), and recombinant *Schistosoma mansoni* antigens (32). Cai et al. generated Tol-DC from murine iPSCs and injected these Tol-DC 7 days before transplantation into the recipients, resulting in a decreased expression of perforin/granzyme B, increased secretion of TGF-β, and proliferation of CTLA4^+^GITR^+^Treg in mice with prolonged cardiac graft survival (73).

## The Function of Tol-DC

**Figure 2:**
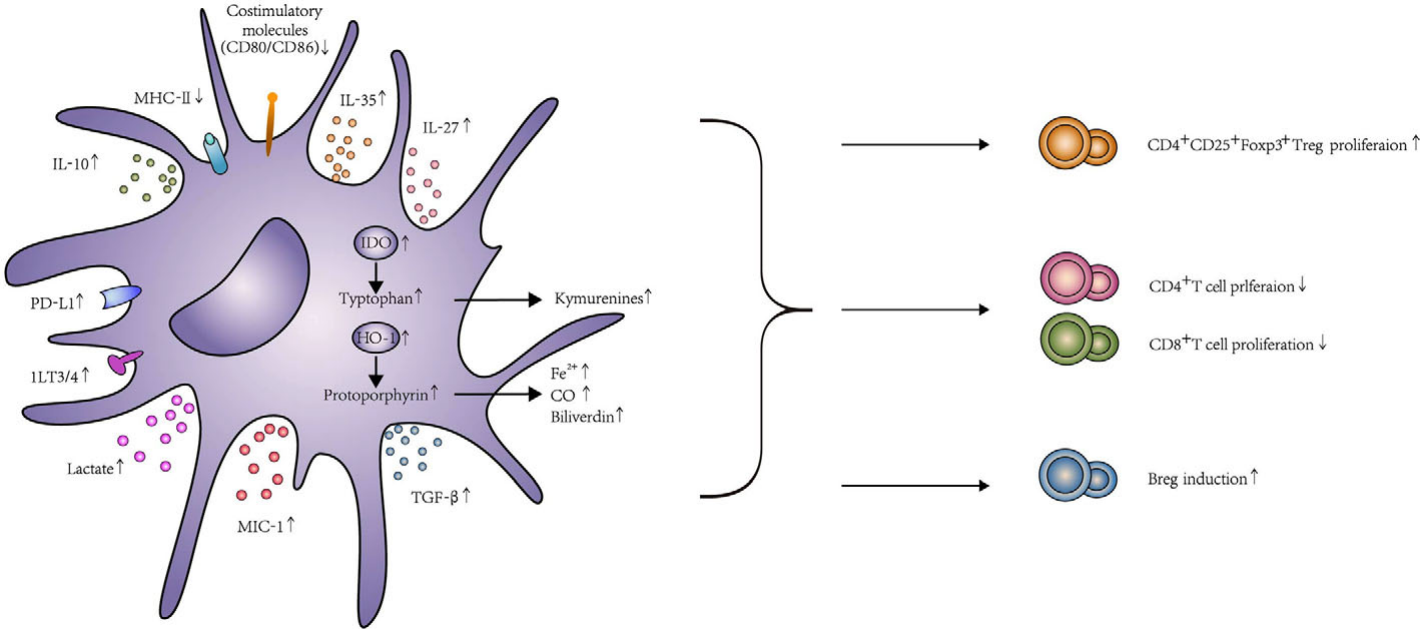
The function and effects of Tol-DC. Tol-DC are characterized by low expression of costimulatory molecules CD80, CD86, and MHC-II. Tol-DC decrease the proliferation of T cells through apoptosis, anergy, and hyporesponsiveness. Meanwhile, they can promote Treg and Breg induction. The mechanism of this process, including contact-dependent and contact-independent mechanisms. Contact-dependent mechanisms include PD-L1, Fas-L, and ILT3/4. Contact-independent mechanisms include the expression of anti-inflammatory molecules, such as IL-10, TGF-β, IL-35, IL-27, and MIC-1. Tol-DC can also exhibit their function through the expression of IDO, HO-1, and lactate. The interaction between PD-1 and PD-L1 delivers inhibitory signals to T cells and contributes to the anergy of T cells. Fas-L expressed on DC can induce T cell apoptosis by combining with Fas expressed on T cells. The increased expression of ILT3/ILT4 in DC contributes to Treg induction. Similarly, IL-10 and TGF-β can broadly inhibit T cell activation by interfering with T cell receptor signaling and eventually promote Treg induction by IDO production. In addition, both IL-27 and IL-35 are considered as important regulators of adaptive immune responses. The high expression of IL-27 was correlated with the induction of IL-10 expressing CD4^+^ T cells, and IL-35 overexpressed DC could increase Treg. DC transfected with MIC expression adenovirus could enhance T cell exhaustion and Treg proliferation. IDO catalyzes tryptophan degradation to form kynurenines, which consequently, impairs T cell proliferation and promotes Treg differentiation. HO-1 catalyzes the conversion of protoporphyrin to biliverdin, Fe^2+^, and CO. HO-1 could promote Treg differentiation and prevent T cell–mediated inflammatory diseases because of the increased CO level. Tol-DC could produce high levels of lactate that shape T cell responses toward tolerance, including declines of glycolysis and activation and proliferation in T cell. In addition to T cell modification, Tol-DC can conditionally induce Breg proliferation, too.

The reason why Tol-DC could become a replacement for IS in future organ transplantation is their ability to decrease T cell proliferation and lead to T cell apoptosis, anergy and hyporesponsiveness. Meanwhile, they also can promote Treg induction to induce the tolerance. These two processes could be summarized as contact-dependent and -independent mechanisms. The contact-dependent mechanism means direct contact between lymphocytes and Tol-DC, which contained surface receptors, such as PD-L1, Fas-L, ILT3, and ILT4. In addition, the contact-independent mechanism means Tol-DC could exert their immunosuppressive ability *via* immunomodulatory molecule release, including immunomodulatory cytokines, such as IL-10 and TGF-β, or enzymes, such as IDO, heme-oxygenase-1 (HO-1), and others. The function of Tol-DC is elucidated in **Figure 2**, and the experimental details are shown in **Table 1**.

**Table 1:** Experimental details of Tol-DC transfer in animal transplant models.

Induction strategy	Phenotype of Tol-DC	Intervention	Transplanted model	Mechanism	Reference No.
DC pretreated with Cobalt Protoporphyrin (COPP)	HO-1^high^MHC-II^low^CD40^low^CD80^low^CD86^low^	Donor-derived Tol-DC(day -7, 5 × 10^6^ i.v.)	Allogeneic mouse cardiac	IFN-γ^+^ T cell↓, alloantibody production↓	(74)
DC infected with Recombinant human growth differentiation factor 15(GDF15) expression adenovirus	GDF15^high^CD40^low^CD80^low^	Autologous Tol-DC (day -7, 1 × 10^6^ i.v.) + Rapamycin (day 0-7,1mg/kg, i.p.)	Allogeneic mouse cardiac	T cell exhaustion↑, CD4^+^ FOXP3^+^Treg↑	(75)
DC treated with recombinant IL-35/Ebi3	MHC-II^low^CD86^low^CD80^low^	Donor-derived Tol-DC (day -1, 1 × 10^5^ i.v.)	Allogeneic mouse cardiac	CD4^+^CD25^+^ FOXP3^+^Treg↑	(76)
DC cultured with urine induced pluripotent stem cells	CD11b^high^CD11c^high^MHC-II^low^CD86^low^CD80^low^	Donor-derived TolDC (day -7, 1 × 10^6^ i.v.)	Allogeneic mouse cardiac	CD4^+^CD25^+^ FOXP3^+^Treg↑, cytotoxic T cell↓, TNF-α↓, IL-1β↓, IL-6↓	(77)
DC2.4 cells transduced with pAd5/F35-GFP-Jagged-1 viruses	Jagged-1^high^MHC-II^intermediate^CD80^intermediate^CD86^intermediate^	Exogenous Tol-DC (day -1, 5 × 10^6^ i.v.)+ anti-CD40L mAb (day 0, 2, 4 and 6, 0.25mg, i.p.)	Allogeneic mouse cardiac	CD4^+^CD25^+^ FOXP3^+^Treg↑, TGF-β↑, IFN-γ↓	(78)
DC infected with Relb shRNA expressing lentivirus, activated by LPS	Relb^low^MHC-II^low^CD86^low^CD80^low^CD83^low^	Donor-derived Tol-DC (day -7, 5 × 10^6^ i.v.)	Allogeneic mouse cardiac	CD4^+^CD25^+^ FOXP3^+^Treg↑	(57)
DC pretreated with LF 15-0195	MHC-II^low^CD86^low^CD40^low^	Exogenous Tol-DC (day -7, 5 × 10^6^ i.v.)	Allogeneic mouse cardiac	CD4^+^CD25^+^CTLA4^+^T cell↑, CD4^+^CD25^+^ FOXP3^+^Treg↑	(56)
DC treated with 0.1ng/ml GM-CSF	CD11c^high^MHC-II^low^CD80^low^CD86^low^	Autologous Tol-DC (day -1, 1 × 10^6^ i.v.) + anti-CD3 Ab (day -1, 300mg, i.v.)	Allogeneic mouse islet	T cell activation↓, alloantibody production↓, CD4^+^ FOXP3^+^Tregs↑	(79)
DCs treated with IL-10	MHC-II^low^CD40^low^CD86^low^CD205^low^IL-12p70^low^TNF-α^low^IL-6^low^IL-10^high^	Autologous Tol-DC (day -1, 2 × 10^6^ i.v.)	Xenogeneic (rat-mouse) islet	Graft-infiltrating CD8^+^CD28^-^ and CD8^+^PD1^+^ suppressor T cell↑	(80)
DCs conditioned with TGF-β, activated by LPS	MHC-II^intermediate^CD80^low^CD86^low^IL-12p70^low^	Donor-derived Tol-DC (day 0, 5× 10^5^ i.v.)	Syngeneic mouse islet	FOXP3^+^Treg ↑	(81)
DCs conditioned with TNF-α and α1-Antitrypsin	MHC-II^low^CD86^low^CD80^low^IL-6^low^IL-12^low^IL-10^high^	Autologous Tol-DC (day 0, 2 × 10^6^ i.v.)	Allogeneic rat kidney	FOXP3^+^Treg↑, TGF-β↑, IL-6↓, IFN-γ↓	(82)
DCs treated with 0.4ng/ml GM-CSF	CD11c^high^MHC-II^low^CD80^low^CD86^low^	Autologous Tol-DC (day -1, 1 × 10^6^ i.v.) + anti-CD3Abs (day −1, 1, 3, 5 and 7, 1mg, i.p.)	Syngeneic mouse skin	CD8^+^ FOXP3^+^Treg↑	(83)
DC cotransfected with plasmids encoding EGFP-hTGF-β1 and EGFP-hFasL	TGF-β^high^Fas-L^high^CD85^low^CD80^low^	Exogenous Tol-DC (day -5, 2× 10^6^ i.v.)	Allogeneic rat liver	IL-10↑, IL-1↓, IL-12↓	(84)
DC treated by GM-CSF,IL-10 and FLT3L	MHC-II^low^CD86^low^CD40^low^CD80^low^	Donor-derived Tol-DC (day -7, 2× 10^6^ i.v.)+Penicillin (day 0, 500u/10g, subcutaneous)	Allogeneic rat kidney	IL-2↓, IFN-γ↓, IL-4↑, IL-10↑, CD4^+^CD25^+^ FOXP3^+^Treg↑	(85)
DCs stimulated by VitD3 and IL-10	CD14^high^MHC-II^low^CD86^low^CD83^low^CD80^low^PD-L1^high^	Donor-derived Tol-DC (day -7 and 3, 5-10× 10^6^ i.v.)+CTLA4 Ig (day -7 and -4, 12.5 mg/kg, day −1, 0, 2, 4, 7 and 10, 20mg/kg i.v.) + Tapered rapamycin maintenance	Allogeneic monkey kidney	CD4^+^CD95^+^Tmem↓, CD8^+^CD95^+^Tmem↓ CTLA4 and PD-1 expressed on Tmem↑	(86)
DCs stimulated by VitD3 and IL-10	CD14^high^MHC-II^low^CD86^low^CD83^low^CD80^low^PD-L1^high^	Donor-derived Tol-DC (day -7 and 3, 5-10× 10^6^ i.v.)+CTLA4 Ig (day -7 and -4, 12.5 mg/kg, day −1, 0, 2, 4, 7 and 10, 20mg/kg i.v.) + Tapered rapamycin maintenance	Allogeneic monkey kidney	Donor-specific Eomes^low^CTLA4^high^CD8^+^ central Tmem↑	(87)
DCs stimulated by VitD3 and IL-10	CD14^high^MHC-II^low^CD86^low^CD83^low^CD80^low^PD-L1^high^	Donor-derived Tol-DC (day -7 and 3, 5-10× 10^6^ i.v.)+CTLA4 Ig (day -7 and -4, 12.5 mg/kg, day −1, 0, 2, 4, 7 and 10, 20mg/kg i.v.) + Tapered rapamycin maintenance	Allogeneic monkey kidney	Donor-specific Eomes^low^CTLA4^high^CD8+ T cell↑, IL-17↓	(88)
DCs stimulated by VitD3 and IL-10	CD14^high^MHC-II^low^CD86^low^CD83^low^CD80^low^	Donor-derived Tol-DC (day -7 and 3, 5-10× 10^6^ i.v.)+CTLA4 Ig (day -7 and -4, 12.5 mg/kg, day −1, 0, 2, 4, 7 and 10, 20mg/kg i.v.) + Tapered rapamycin maintenance	Allogeneic monkey kidney	Donor-Specific CD4^+^CTLA4^high^ T Cell proliferation	(89)

### Contact-Dependent Mechanism

PD-1 is an important inhibitory molecule expressed on T cells, and PD-L1 is its ligand expressed on DC. The interaction between PD-1 and PD-L1 delivers inhibitory signals to T cells and contributes to the anergy of T cells (90). According to recent literature, cross-dressed DC in the graft are characterized by high expression of PD-L1 after murine liver transplantation, and these cross-dressed DC failed to stimulate proliferation of allogeneic T cells but markedly suppressed antidonor host T cell proliferation *in vitro* (91). DC transfected with PD-L1 recombinant adenovirus could prolong the survival in rat renal transplantation. The effect is correlated with the suppression of CD8+T cell and the decreased secretion of proinflammatory cytokines (92). Fas and Fas-L belong to the TNF receptor and ligand family, respectively. Fas-L expressed on DC can induce T cell apoptosis by combining with Fas expressed on T cells (93). Mono-DC cotransfected with TGF-β1/Fas-L could prolong the survival time in murine liver transplantation. The increased level of Fas-L could induce T cell apoptosis (84). Immature DC transduced by lentiviral vectors expressing human IL-10 and FasL genes could significantly reduce the expression of costimulatory molecules and T cell proliferation and extend the survival period of rat liver allografts (94). Tol-DC have a unique subset: CD11b^high^Ia^low^ Tol-DC. They can express Fas and inhibit T-cell proliferation in a negative feedback manner through increased IL-10 levels (49). ILT3 and ILT4 belong to inhibitory receptors, which can modulate IκB phosphorylation and degradation through SH2 domain-containing protein tyrosine (SHP) phosphatases, inhibit the activation of NF-κB, and induce Tol-DC phenotype (95). The number of ILT3/ILT4^+^ DC in patients who received long-term rapamycin after renal transplantation is significant increased. The increased ILT3/ILT4^+^ DC contributed to Treg induction and expansion of CD8^+^CD28^-^T cell (96).

### Contact-Independent Mechanism

IL-10 has always been considered a powerful anti-inflammatory molecule in different diseases (97). IL-10 not only inhibits T cell proliferation, but also shows the ability to induce Treg. Tol-DC induced by IL-10 could also release high levels of IL-10. Prolonged xenograft survival of rat islets in diabetic mice was observed after an autologous IL-10-pulsed DC administration without any immunosuppressive treatment. The injection of IL-10-pulsed DC enriches graft infiltrating regulatory CD8^+^T cells and tolerogenic myeloid cells with suppression-associated phenotypes (80). DC cotransfected Fas-L and IL-10 have more capacity to inhibit T cell activation and prolong the survival period of allografts than Fas-L alone (94).

TGF-β plays a pivotal role in transplant tolerance, which broadly inhibits T cell activation by interfering with T cell receptor signaling and eventually promotes Treg induction by IDO production (98). If there is a decrease of the expression of TGF-β2 receptors on DC, both T and B cell activation and reduction of the expression of Foxp3 in Treg would occur (99). Smad7 is a potent negative regulator of TGF-β signaling. The presence of Smad7 could prevent the binding of Smad2 and Smad3 to the TGF-β2 receptor, and this inhibitory effect is essential for TGF-β signal transduction. Rodent DC derived from Smad7 deficiency are resistant to the development of experimental autoimmune encephalomyelitis (EAE) due to an increase of protective Treg and inhibition of encephalitogenic effector T cells in the central nervous system (100). TGF-β gene modified DC exhibit the immature phenotype with low expression of MHC-II, CD80, CD86, and CD40, which could downregulate antigen presentation of bone morrow-derived immature DC. The high expression of TGF-β inhibits T cell proliferation and delays the progress of murine inflammatory bowel disease (IBD) (101). Tol-DC generated from TGF-β increase the frequency of Tregs in islet graft and shows long-term graft survival (102). DC cotransfected with plasmids encoding TGF-β and FasL show low expression of CD85 and CD80. These Tol-DC decrease Banff rejection activity index and allow graft function recovery in rat liver grafts, which is correlated to the increased expression of IL-10 and decreased expression of IL-1 and IL-12 (103).

In addition to classical immunomodulatory molecules, such as IL-10 and TGF-β, there are also other cytokines released from Tol-DC, which could regulate T cell activation and Treg proliferation. IL-35 and IL-27 are the members of IL-12 family. Both IL-27 and IL-35 are considered important regulators of adaptive immune responses (104). Under LPS stimulation, mono-DC secrete high levels of IL-35 to prevent the maturation of DC. IL-35 could activate STAT3 and STAT4 signal pathways in DC. On day 1 prior to transplantation, IL-35 overexpressed DC could increase IL-10 and Treg levels in cardiac recipients and lead to prolonged allograft survival (76). IL-27 is mainly produced by DC stimulated by microbial products or other immune stimuli. IL-27 could promote the differentiation of Th1 and type 1 regulatory (Tr1) cells but inhibit Th2 and Th17 cells (105). Overexpression of IL-27 combined with the application of rapamycin could definitely improve cardiac allograft acceptance. The high expression of IL-27 is also correlated with the induction of IL-10 expressing CD4^+^T cells (106). Moreover, DC transfected with MIC expression adenovirus could enhance T cell exhaustion and Treg proliferation and consequently promote the survival of cardiac allograft (100, 107).

IDO is known to act as a bridge between DC and Treg. IDO catalyzes tryptophan degradation to form kynurenines and consequently impairs T cell proliferation and promotes Treg differentiation (108). Most Tol-DC are characterized by high expression of IDO. Before rat renal transplantation, recipient rats were preinjected with autologous Tol-DC treated with donor alloantigens. The renal allograft exhibited a lighter rejection response and longer graft survival time. This remission was thought to be correlated with increased Treg. However, when IDO is silenced by siRNA in rats, the rejection response is aggravated (85). α1-Antitrypsin is a circulating glycoprotein. α1-Antitrypsin-pulsed DC are characterized by decreased expression of MHC-II, CD80, and CD86 and high expression of IDO. After transferring these IDO^+^ Tol-DC, the kidney allograft survival period is prolonged and Treg increase (87). Human soluble CD83 (hsCD83) is able to inhibit DC maturation and cause the anergy of Teff. In both heart and renal transplant models, the injection of hsCD83 down-modulates the expression of costimulatory molecules and up-modulates IDO in DC, which can prolong the allograft survival period (109, 110).

HO-1 is an enzyme that catalyzes the conversion of Fe-Protoporphyrin-IX (Heme group) to biliverdin, ferrous ion, and carbon monoxide (CO). HO-1 could promote Treg differentiation (111) and prevent T cell–mediated inflammatory diseases because of the increased CO level (112). CO can reduce both mitochondrial membrane potential and ATP production, which results in mitochondrial dysfunction in DC. The high expression of HO-1 in DC can resist LPS-induced maturation and release high levels of IL-10. HO-1 expressing DC could modulate the severity of lung inflammatory responses in murine models of airway inflammation with increased Treg (111). Cobalt protoporphyrin (CoPP) is the agonist of HO-1, and DC treated with CoPP are characterized by high expression of HO-1. Adoptively transferring donor-derived high HO-1 expressing immature DC 7 days before transplantation effectively blocks the activation of both T and B cells in cardiac allograft mice (74).

In addition to IDO and HO-1, NO, PGE2, and adenosine also exhibit great capacity to induce Tol-DC. Chloroquine (CQ), an antimalarial drug, also induces Tol-DC and, consequently, promotes the expression of NO synthase and, finally, results in the inhibition of T cell activation (113). After transferring CQ-pulsed DC to EAE mice, a decline of glial reactivity in the central nervous system is observed (114).

### Breg Induction

In addition to T cell modification, Tol-DC can conditionally induce Breg proliferation. When Tol-DC are administered to nonobese diabetic (NOD) mice, two tolerogenic B-cell subsets, CD19^+^B220^+^CD11c^−^IL-10^+^ B cell and B10 cell proliferate (115). Breg could proliferate through the retinoic acid receptor, which combines with retinoic acid released from Tol-DC (115, 116). The remission of IBD in the mouse model after administrating monocyte derived Tol-DC is correlated with the induction of IL-10-Bregs. However, whether Breg could be induced by Tol-DC in transplantation models or not remains to be further explored.

## The Metabolism Modification of Tol-DC

### General Metabolism in Tol-DC

Glycolysis is an indispensable metabolic process in our body, which can rapidly decompose glucose into ATP and supply energy (117). LPS, an agonist of TLR4, is widely used to induce functional DC. However, during this process, DC activation relies on glycolysis for abundant ATP (118). Citrate is a tricarboxylic acid (TCA) cycle intermediate, which plays an important role in LPS-induced DC activation. LPS activates TLR and, consequently, causes glycolysis inside DC through the generation of citrate and the synthesis of fatty acids *in vivo*, which could promote the expansion of endoplasmic reticulum and Golgi networks required for DC activation (119). Complement component C1q subcomponent-binding protein (c1qbp), a multifunctional chaperone protein, plays an important role in mitochondrial function and supports mitochondrial metabolism and DC maturation. The production of citrate regulates DC maturity *via* c1qbp-dependent pyruvate dehydrogenase activity (120). 2-deoxyglucose impairs glycolysis in DC, which contributes to the decreased expression of CD40, CD86, and MHC-II and production of IL-6, IL-12p70, and TNF and causes a Tol-DC phenotype (119). The decline of glycolysis in DC could contribute to impaired maintenance of dendritic shape, motility, CC-chemokine receptor (CCR)7 oligomerization, and migration to draining lymph nodes (121). In malignant melanoma, paracrine-derived Wnt5a protein can alter the metabolic pathway of DC by stimulating β-catenin signaling pathway, which can shift local DC populations from a glycolytic state to oxidative phosphorylation (OXPHOS) and fatty acid oxidation (FAO) *via* peroxisome proliferator-activated receptor (PPAR)-γ-carnitine palmitoyl transferase-1 (CPTIA) axis (122). In a recent first-in-human clinical trial of kidney transplantation, Marin et al. report that autologous Tol-DC could produce high levels of lactate that shape T cell responses toward tolerance, including declines of glycolysis, activation, and proliferation in T cell (123).

During the induction of VitD3-pulsed Tol-DC, genes related to OXPHOS and the protein O-linked glycosylation pathway are overexpressed (23). 1,25-dihydroxy vitamin D_3_ is the active form of vitamin D, which can induce human monocyte-derived Tol-DC by metabolic reprogramming and upregulate several genes directly correlated to glucose metabolism, TCA, and OXPHOS (124). As discussed above, Dex has already been applied to induce Tol-DC in clinical practice. García-González et al. studied the transcriptional profile of mono-DC from healthy donors modulated with Dex and activated with monophosphorylate lipid A (MPLA), demonstrating that genes related to FAO are strongly enriched, predicting the activation of alternative metabolic processes than those driven by the counterpart DC (125). Increased expression of inducible nitric oxide synthase (iNOS) (126) and inhibition of 5’ adenosine monophosphate-activated protein kinase (AMPK) (127) decreased OXPHOS and FAO in immunologic DC. The activation of nuclear factor E2-related factor 2 (Nrf2) can inhibit the production of iNOS, thereby restoring OXPHOS as the energy source in Tol-DC (128). Compared to immunologic DC, Tol-DC possess a steady OXPHOS program and favors FAO (129). FAO has a regulatory effect on OXPHO. Fatty acids can suppress the TLR-induced hexokinase activity and perturb tricarboxylic acid cycle metabolism, which enhances the production of mitochondrial reactive oxygen species (ROS) (130). miR-142 is central to metabolic reprogramming. Sun et al. demonstrated that miR-142 directly targets carnitine palmitoyltransferase-1a, a key regulator of the fatty acid pathway to regulate FAO. In miR-142 deficient mice, DC fail to shift from OXPHOS to glycolysis and show reduced production of proinflammatory cytokines and ability to activate T cells *in vitro* and *in vivo* models of sepsis and allogeneic immunity (131).

The production of ROS is proven to be more likely related with the immunogenic DC. α-Glucans in *Mycobacterium tuberculosis* can induce ROS production and lead to DC maturation and lymphocyte proliferation, which is partly related to the induction of spleen tyrosine kinase (Syk) (132). The reduction in mitochondrial ROS production dramatically decreases the cross-presentation capacity of pDC and strongly impairs their ability to trigger CD8^+^T cell responses (133). Mogilenko et al. also report that reducing mitochondrial ROS production in DC ameliorates the disease in an IL-23-dependent model of psoriasis because of the reduction in IL-23 and skin inflammation (130).

**Figure 3:**
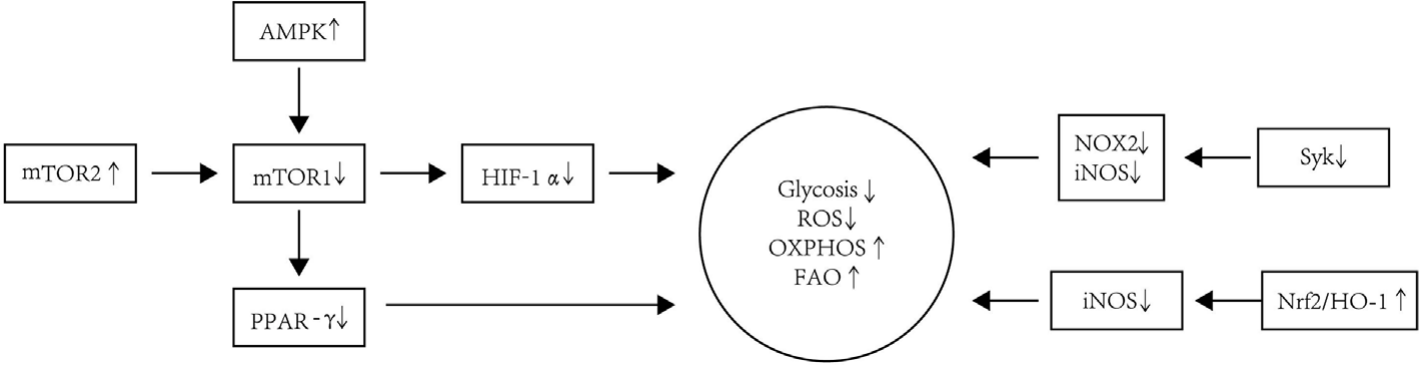
The metabolism modification of Tol-DC. Tol-DC are usually characterized by increased OXPHOS and FAO but decreased ROS and glycolysis. The inhibition of mTOR is correlated with the tolerogenic metabolism in DC. AMPK is one of the main protein kinases regulating glucose metabolism and is located upstream of the mTOR1. The increased expression and activation of AMPK decrease the expression and activation of AMPK downstream kinase mTOR1. The PPAR-γ is the downstream target of mTOR1. The inhibition of mTOR1 can also decrease expression of PPAR-γ, which is a response to lipid metabolism in DC. HIF-1α is responsible for sustained glycolytic reprogramming in DC. The blockage of mTOR1 can influence the expression of HIF-1α. mTOR2 can inhibit mTOR1-regulated metabolic function in DC. Additionally, the blockade of Syk signaling leads to a decrease in levels of iNOS and NOX2, which contributes to the decreased glycolysis and ROS in DC. Nrf2/HO-1 can inhibit the production of iNOS, thereby restoring OXPHOS as the energy source in Tol-DC.

In summary, Tol-DC is usually characterized by increased OXPHOS and FAO but decreased ROS levels. The phenotype and metabolism of Tol-DC are summarized in **Figure 3**.

### mTOR and Tol-DC Metabolism

mTOR is known to be divided into two complexes: mTOR complex 1 (mTORC1) and mTORC2. The differentiation of DC induced by GM-CSF and IL-4 from human monocytes relies on the mammalian target of mTORC1 activation *via* phosphoinositide 3-kinase (134). mTORC1 pathway has a central role in the pathogenesis of some autoimmune diseases and is a mediator of the Warburg effect that allows cell survival under hypoxia (135). Rapamycin, an mTOR blocker, has been widely used to prevent rejection after organ transplantation. Rapamycin-induced DC administration is shown to play an immunosuppressive role in skin transplantation (41). Polymerized allergoids conjugated to mannan (PM), which can induce the tolerance of DC, are thought to be vigorous vaccines for allergen-specific immunotherapy. However, when PM-pulsed DC are adsorbed to alum, their capacity to generate Treg is impaired. This phenomenon is related to the inhibition of mTOR by alum, which alters metabolic reprogramming by transforming glycolytic pathways and inhibiting ROS production in PM-pulsed DC (136). PPAR-γ is the downstream target of mTORC1, which is upregulated early in mono-DC differentiation, affecting mono-DC maturation and function largely through control of lipid metabolism (137). The relationship between the mTOR signaling pathway and metabolism may involve multiple mechanisms. Activation of the mTOR signaling pathway can stimulate hypoxia-inducible factor-1α (HIF-1α) (138), which is responsible for sustained glycolytic reprogramming in DC (121). In HIF-1α knockout mice, APC express lower levels of MHC-II and costimulatory molecules and are less able to induce T-cell proliferation (139). Graphene quantum dots (GQD) are atom-thick nano-dimensional carbon sheets with excellent physico-chemical and biological properties. GQD promote tolerogenic functions in mono-DC, which prevent the pathologies caused by inflammatory T cells. This process is mediated by the reduced activity of mTOR by GQD, which is correlated to the increase in transcription of autophagy genes and autophagic flux in DC (140). AMPK is one of the main protein kinases regulating glucose metabolism and is located upstream of the mTOR. Polyphenol resveratrol is an antitumor drug that has been used in clinical trials and can increase the expression and activation of AMPK and caspase-3 and decrease the expression and activation of AMPK downstream kinase mTOR (141).

Moreover, mTOR1 and mTOR2 can also affect each other. mTORC2 can inhibit mTORC1-regulated metabolic function in DC. mTORC2 knockout DC improves mTORC1 metabolic activity, which is biased toward glycolytic metabolism to generate ATP, increased lipid content, and higher viability stimulated by LPS. Enhanced integrin alpha IIb (Itga2b) and protein kinase 2 (Ptk2)/focal adhesion kinase (FAK) expression can activate hematopoietic cell signal transducer expression and enhance mTORC1 activity (142).

In conclusion, mTOR is important in the metabolism modification of Tol-DC. The inhibition of mTOR could induce the tolerogenicity in DC. The prevention of mTOR activation could contribute to the transformation of OXPHOS and decreased production of ROS. In the mTOR signaling pathway, HIF-1α is responsible for sustained glycolytic reprogramming, and PPAR-γ controls lipid metabolism in DC.

### Syk and Tol-DC Metabolism

In addition to the mTOR signaling pathways, Syk can also play an important role in DC metabolism. The activation of Syk contributed to the sustained glycolytic reprogramming in DC. Other than TLRs, C-type lectin receptors (CLRs) are also expressed on DC as PRR to recognize pathogen-associated stimuli, such as dectin-1/2. Fungal-associated β-glucan ligands react with dectin1/2 and induce glycolytic reprogramming in DC *via* a Syk-dependent way, which contributes to the production of IL-1β (143). Dectin-1 binding with annexins which is expressed on apoptotic cells induce a tolerogenic DC phenotype. This is a distinct mechanism from that of the interaction site of pathogen-derived β-glucans and induces selective phosphorylation of Syk, causes activation of nicotinamide adenine dinucleotide phosphate (NADPH)-oxidase-2 (NOX2), moderates production of ROS, (144). The blockade of Syk signaling leads to the improvement of sepsis-induced acute kidney injury in mice as suggested by the attenuation of creatinine/blood urea nitrogen in serum, renal myeloperoxidase activity, and repair of tubular structures in the kidney. This can be correlated to a decrease in levels of IL-6/MCP-1 in CD11c^+^DC and iNOS, NOX2, and nitrotyrosine in neutrophils (145). Syk signaling may serve as an effective therapeutic target in innate immune cells to limit inflammatory cascade, and the inhibition of Syk might prevent glycolysis in DC and lead to the tolerogenicity of DC.

## PreClinical and Clinical Attempts of Tol-DC in Organ Transplantation

Autoimmune disease is a series of dysfunctions and tissue damage caused by the loss of tolerance to self-antigen. Clinical trials are currently carried out to explore the efficacy and safety of transferred Tol-DC to treat autoimmune diseases such as type 1 diabetes mellitus (T1DM), multiple sclerosis (MS), rheumatoid arthritis (RA), and CrD (146). Currently, clinical trials on Tol-DC are in a preliminary stage. What has been proven so far is the safety of Tol-DC to the human body. The efficacy of Tol-DC has a close association with the increase in Treg levels. Further studies are needed to explore the optimal strategies of Tol-DC application in clinical practices. However, there is still no study reporting the efficacy of Tol-DC in human transplantation, so we would like to discuss the efficacy of Tol-DC in nonhuman primate renal transplantation and the current registered clinical trials of Tol-DC related to organ transplantation in clinicaltrials.gov.

### Tol-DC in Nonhuman Primate Kidney Transplantation

In recent years, the study of Tol-DC for kidney transplantation has improved. The first preclinical trial of Tol-DC in renal transplantation showed that donor-derived Tol-DC induced by VitD3 and IL-10 were characterized by low expression of CD80 and CD86 and high levels of PD-L1. Tol-DC were cotransferred into rhesus monkey recipients before renal transplantation, with a combined application of CTAL4Ig [blocker of CD80/86 (147, 148)] and rapamycin and without CNI and steroids, which showed Tol-DC injection could prolong the survival of grafts in monkeys (86). CD95 (Fas)^+^ T cells are considered to have memory capacity, which includes central (CD28^+^) and effector memory (CD28^-^) T cells in rhesus monkey. Both PD-1 and CTLA4 are considered markers of exhaustion and expressed on rhesus CD95^+^ T cell. The administration of Tol-DC could shift CD95^+^ Tmem to an immunosuppressive phonotype with increased expression of PD-1 and CTLA4 (86). Thereafter, they further explored the mechanism of prolonged graft survival after administration of donor-derived Tol-DC. Eomesodermin (Eomes), a key transcription factor in CD8^+^ Tmem (149), play a critical role in long-term survival of antigen-specific central Tmem. The prolonged survival of renal allografts after both CTAL4Ig and donor-derived Tol-DC therapy might be related to the maintenance of donor-reactive Eomes^low^CTLA4^high^ central Tmem, which displayed a regulatory phenotype *in vivo* (87). Compared to CNI, CTLA4Ig may preserve renal function and improve long-term outcomes in kidney transplantation (150). The same research team found the infusion of CTLA4Ig and Tol-DC together could maintain the expression of CTLA4 in CD4^+^ T cells in another similar preclinical trial. The exposure of CTLA4-expressed CD4^+^ T cells to donor antigens is essential for the prevention of Teff responses and the promotion of transplant tolerance (89). In addition to donor-derived Tol-DC, the effect of autologous Tol-DC is also evaluated in a preclinical trial of rhesus monkey. Autologous Tol-DC are incubated with vesicles generated from prospective transplant donor PBMC, and these Tol-DC could effectively capture vesicles without changing their own phenotype (88). IL-17 is a proinflammatory cytokine, which plays an important role in organ rejection. The deficiency or neutralization of IL-17 is protective against the development of kidney allograft rejection (151). After transplantation, there was an increased absolute number of donor-reactive CD4^+^IL-17^+^ T cells in the renal allograft of rhesus monkey in nondonor antigen-pulsed autologous Tol-DC treated group. However, the number of donor-reactive CTLA4^+^IL-17^+^ T cells did not change pre- and post-transplantation in the donor antigen-pulsed autologous Tol-DC treated group. In addition to the inhibition of donor-reactive CTLA4^+^IL-17^+^ T cells, donor antigen-pulsed autologous Tol-DC also modulated the expression of PD-1 and CTLA4 in donor reactive T cells (88). In conclusion, the efficacy of Tol-DC in preclinical trials of kidney transplantation has been proven. By administrating either the donor-derived Tol-DC or donor-antigen pulsed autologous Tol-DC, the survival time of the grafts is prolonged. The prolonged survival of the graft is correlated with the increased expression of PD-1 and CTLA4 and the decreased expression of Eomes in donor-reactive T cells. Meanwhile, the administration of Tol-DC can modulate IL-17-mediated inflammation in renal transplantation.

Tol-DC induced by VitD3 and IL-10 could maintain a stable state both *in vivo* and *in vitro*. Even stimulated by inflammatory molecules, Tol-DC are fully resistant to phenotypic maturation *in vitro* (152). Rhesus T cells stimulated initially with Tol-DC failed to proliferate following restimulation with donor alloreactive antigen in a secondary mixed leukocyte reaction, which ensures the stability of Tol-DC injection *in vivo* (86). Compared to non-Tol-DC treated group, the administration of donor-derived Tol-DC significantly prolonged the graft survival period ranging from 50 to 300 days (median=113.5). Graft median survival time of donor-antigen-pulsed autologous Tol-DC was 56 days (88). Additionally, there was no adverse effect observed in these preclinical trials. Meanwhile, the injection of Tol-DC could not induce the circulating donor-specific allogenic antibody, which indicated that Tol-DC could function stably for a long time in the body (86). However, further clinical studies are needed to address the safety, stability, and feasibility of Tol-DC transfusion in human transplantation.

### Administration Route and Migration of Tol-DC in Organ Transplantation

Although Tol-DC has been proven effective in rodent and rhesus monkey organ transplantation, it is also important to explore the best administration route. The administration route not only influences the effect of Tol-DC but also the migration of Tol-DC *in vivo*. In an experimental autoimmune encephalomyelitis (EAE) model, intraperitoneal (i.p.) administration of Tol-DC could effectively suppress clinical manifestation of ongoing experimental autoimmune myasthenia gravis more than intravenous (i.v.) administration by regulating T and B cell responses (153). In clinical trials of Tol-DC that have been reported so far, administration routes of intradermal (i.d.) (154, 155), i.p. (156) and i.v. (157) were all proven to be safe and well tolerated in human. However, i.v. administration of autologous Tol-DC was proven to have better immune tolerance than i.d. in rhesus monkeys (158). Another report demonstrated that 1 day after i.v. injection of Tol-DC in rat liver transplantation, the number of administrated Tol-DC was the highest in the liver graft and also detected in other second lymphoid organs. However, when it came to i.p. administration, the number of Tol-DC was the highest in abdominal lymph nodes 24–48 h after injection, but there were few in the rat liver graft (84). The result implicates that i.v. injection of Tol-DC is preferred to migrate to the graft than i.p. In **Table 1**, we find i.v. injection is the most commonly used route for Tol-DC administration in animal transplant models. In addition, the i.v. route is more readily operated in the clinical practice. Taken together, we recommended i.v. to be the best administration route of Tol-DC injection in future human clinical attempts at transplantation.

The migration of Tol-DC is not only influenced by administration routes, but also by the expression of chemokine and its receptors. Immature DC are characterized by high expression of CCR2, CCR5, and CCR6 and access to nonlymphoid tissues through attraction of CC-chemokine ligand (CCL)2, CCL5, and CCL21, whereas mature DC are characterized by high expression of CCR7, which allows DC to recognize the lymph node-directing chemokines CCL19 and CCL21 (159). Tol-DC tend more to a semimature state. Tol-DC induced by Dex and Vitd3 express chemokine receptors characteristic of an immature phenotype, such as CCR2, CCR5, CXCR1, and CXCR2. However, under stimulation by LPS, Tol-DC downregulates the expression of these chemokine receptors and upregulates the expression of CCR7 although the level of expression is lower than activated DC. The stimulation of LPS induces Tol-DC to migrate in response to CCL19 and move to the lymph nodes (160). Although using a model of allotolerance induction, Liu et al. show a striking failure to tolerate cardiac allografts in CCR7-deficient recipients. The deficiency of CCR7 contributed to a significantly reduced number of pDC in peripheral as well as mesenteric lymph nodes. After single transfer of syngeneic wild-type pDC, the result of cardiac transplantation in CCR7-deficient recipients has significantly improved in a dose-dependent manner (161). This report demonstrates pDC with high expression of CCR7 is considered as a kind of Tol-DC in transplant models. Additionally, α-1 antitrypsin (AAT) is reported to induce the tolerance of DC, and the upregulation of CCR7 is observed in AAT-induced Tol-DC stimulated by inflammatory molecules. The expression of CCR7 induced Tol-DC to migrate to draining lymph nodes in an islet transplantation model (162). In conclusion, Tol-DC expressed a relatively low level of CCR7. However, under the external stimulus, Tol-DC could upregulate the expression of CCR7 and migrate to the second lymphatic organ to induce the anergy of T cells.

### Registered Clinical Trials of Tol-DC in Human Organ Transplantation

The first Tol-DC clinical trial in living-donor renal transplantation has been performed to evaluate the safety of administering autologous Tol-DC (NCT02252055) and is still ongoing. Another phase I clinical trial on Tol-DC in living-donor renal transplantation is recruiting (NCT03726307) currently. Its purpose is to evaluate the safety and feasibility of a single infusion of donor-derived Tol-DC administration 7 days before transplantation and explore the best injection dose. Participants will be maintained on a triple immunosuppressant scheme with mycophenolate mofetil, tacrolimus, and prednisone. Additionally, a Tol-DC clinical trial for liver transplantation is being enrolled (NCT04208919). Tol-DC in living donor liver transplantation phase I/II will be evaluated for safety and therapeutic effect a week after Tol-DC infusion, and immunosuppression weaning will be initiated. The levels of donor special antigen and the change in renal function, quality of life, and cardiovascular risk factors will be used as indicators of evaluation. The effect of Tol-DC-based treatment on the prognosis of organ transplantation is still being evaluated, and the clinical attempts of Tol-DC therapy are still in Phase I and II clinical trials.

## Conclusion and Future Prospective

Organ transplantation is thought to be the most commonly used treatment for end-stage visceral diseases. However, the rejection after operation seriously affects the prognosis of patients. Although the application of IS effectively prolongs the survival of patients, the side effects of IS also influence the life quality of patients. Tol-DC are a small part of DC. They are characterized by low expression of costimulatory moles and proinflammatory factor. Tol-DC induce immune tolerance by inhibiting the activation of T cells and inducing Treg proliferation. There are various agents that can induce the tolerance of DC. These agents include anti-inflammatory cytokines, antisense oligonucleotides targeting costimulatory molecules, IS drugs, VitD3, and PGE2, and so forth. However, there is still no consensus as to the optimal protocol to be used for generation of clinical-grade Tol-DC. More efficient induction protocols remain to be explored in the future. There is growing evidence proving that distinct metabolic reprogramming acts as a regulatory switch in determining the diversity of DC. Tol-DC possess a prominent and stable OXPHOS program and favor FAO but decreased ROS. The targets for the metabolism of Tol-DC are promising tools for tolerogenic vaccination in the future clinical practice. At present, several clinical trials of Tol-DC have been reported. The safety and effectiveness of Tol-DC have been evaluated. However, clinical trials of Tol-DC have stayed in the elementary stage. Future studies are required to identify the optimal dose of Tol-DC and the mechanism of the efficacy. There is still no published report on clinical trials using tolerogenic DC vaccines in organ transplantation. However, the preclinical trials of Tol-DC have been reported. The effect of Tol-DC in organ transplantation is associated with the induction of Treg in rhesus monkey. A phase 1 clinical trial for Tol-DC in organ transplantation is still under recruitment. It will provide valuable insights into the value of these regulatory immune cells for improved prognosis in organ transplantation.

## Author Contributions

QZ and HC collected the literatures and drafted the initial manuscript. YM and QZ revised the manuscript and edited the language. YM conceptualized and guaranteed the review. QC and ZL designed the figures and tables. SL formatted the references and whole manuscript. All authors contributed to the article and approved the submitted version. QZ and HC contributed to this paper equally.

## Funding

This study was supported by grants from the National Natural Science Foundation of China (81700658), the Hunan Provincial Natural Science Foundation-Outstanding Youth Foundation (2020JJ3058).

## Conflict of Interest

The authors declare that the research was conducted in the absence of any commercial or financial relationships that could be construed as a potential conflict of interest.
